# Elevating the uses of storytelling approaches within Indigenous health research: a critical and participatory scoping review protocol involving Indigenous people and settlers

**DOI:** 10.1186/s13643-020-01503-6

**Published:** 2020-11-04

**Authors:** Kendra L. Rieger, Sarah Gazan, Marlyn Bennett, Mandy Buss, Anna M. Chudyk, Lillian Cook, Sherry Copenace, Cindy Garson, Thomas F. Hack, Bobbie Hornan, Tara Horrill, Mabel Horton, Sandra Howard, Janice Linton, Donna Martin, Kim McPherson, Jennifer Moore Rattray, Wanda Phillips-Beck, Rebecca Sinclair, Annette S. H. Schultz

**Affiliations:** 1 School of Nursing, Trinity Western University, Langley, Canada; 2 Lakota First Nation; Professional and French Language Services, Manitoba Teachers Society, Winnipeg, Canada; 3 Faculty of Social Work, University of Manitoba, Winnipeg, Canada; 4 Max Rady College of Medicine, Rady Faculty of Health Sciences, University of Manitoba, Northern Connections Medical Center, Winnipeg, Canada; 5 Department of Family Medicine, Rady Faculty of Health Sciences, University of Manitoba, Winnipeg, Canada; 6 Sagkeeng First Nation, Canada; 7 Ojibways of Onigaming First Nation, Canada; 8 Interlake Reserves Tribal Council, Headingley, Canada; 9 College of Nursing, Rady Faculty of Health Sciences, University of Manitoba, Winnipeg, Canada; 10 Pimicikamak First Nation, Canada; 11 College of Nursing, Rady Faculty of Health Sciences, University of Manitoba, Winnipeg, Canada; 12 Nisichawayasihk Cree Nation, Canada; 13 Northern Health Region, Flin Flon, Manitoba Canada; 14 Neil John Maclean Health Sciences Library, Bannatyne Campus, University of Manitoba, Winnipeg, Canada; 15 College of Nursing, Rady Faculty of Health Sciences, University of Manitoba, Winnipeg, Canada; 16 Southern Chiefs’ Organization, Winnipeg, Canada; 17 First Nation Health and Social Secretariat of Manitoba, Indigenous Research Chair in Nursing, University of Manitoba, Winnipeg, Canada; 18 Little Saskatchewan First Nation, Canada; 19 College of Nursing, Rady Faculty of Health Sciences, University of Manitoba, Winnipeg, Canada

**Keywords:** Storytelling, Stories, Indigenous peoples, First Nations, Inuit, Metis, Aboriginal and Torres Strait Islander peoples, Maori, Aboriginal peoples, Native Americans, Alaska Native peoples, Two-eyed seeing, Decolonizing research, Qualitative, Scoping review, Health, Turtle Island, Canada, USA, Australia, Aotearoa, New Zealand

## Abstract

**Background:**

There is a complicated and exploitative history of research with Indigenous peoples and accompanying calls to meaningfully and respectfully include Indigenous knowledge in healthcare. Storytelling approaches that privilege Indigenous voices can be a useful tool to break the hold that Western worldviews have within the research. Our collaborative team of Indigenous and non-Indigenous researchers, and Indigenous patients, Elders, healthcare providers, and administrators, will conduct a critical participatory, scoping review to identify and examine how storytelling has been used as a method in Indigenous health research.

**Methods:**

Guided by two-eyed seeing, we will use Bassett and McGibbon’s adaption of Arksey and O’Malley’s scoping review methodology. Relevant articles will be identified through a systematic search of the gray literature, core Indigenous health journals, and online databases including Scopus, MEDLINE, Embase, CINAHL, AgeLine, Academic Search Complete, Bibliography of Native North Americans, Canadian Reference Centre, and PsycINFO. Qualitative and mixed-methods research articles will be included if the researchers involved Indigenous participants or their healthcare professionals living in Turtle Island (i.e., Canada and the USA), Australia, or Aotearoa (New Zealand); use storytelling as a research method; focus on healthcare phenomena; and are written in English. Two reviewers will independently screen titles/abstracts and full-text articles. We will extract data, identify the array of storytelling approaches, and critically examine how storytelling was valued and used. An intensive collaboration will be woven throughout all review stages as academic researchers co-create this work with Indigenous patients, Elders, healthcare professionals, and administrators. Participatory strategies will include four relational gatherings throughout the project. Based on our findings, we will co-create a framework to guide the respectful use of storytelling as a method in Indigenous health research involving Indigenous and non-Indigenous peoples.

**Discussion:**

This work will enable us to elucidate the extent, range, and nature of storytelling within Indigenous health research, to critically reflect on how it has been and could be used, and to develop guidance for the respectful use of this method within research that involves Indigenous peoples and settlers. Our findings will enable the advancement of storytelling methods which meaningfully include Indigenous perspectives, practices, and priorities to benefit the health and wellbeing of Indigenous communities.

**Systematic review protocol registration:**

Open Science Framework (https://osf.io/rvf7q)

## Background

In the current era of reconciliation, health research that prioritizes Indigenous values, epistemology, and histories has become imperative and researchers are increasingly exploring decolonizing research agendas [1]. Decolonizing research approaches shift the nature of relationships, with the aim to build relationships of mutual trust and respect [1]. In Canada, the Tri-Council Policy Statement [2, 3] clearly outlines that research concerning Indigenous peoples must be respectful, involving engagement and collaboration and resulting in reciprocal relationships. Within Indigenous health research, storytelling has emerged as an approach that acknowledges Indigenous oral traditions, creates spaces to share wholistic knowledge about health and illness experiences, and invites community involvement [4, 5]. As a research method, storytelling privileges the voices of those often marginalized and silenced within society; thus, it can be a powerful decolonizing approach [4].

Given the historical legacy, and ongoing realities of health research as a colonial tool that delegitimizes Indigenous peoples’ knowledge and ways of being, a critical and participatory scoping review [6, 7] of how storytelling is being used within Indigenous health research studies is essential. Our diverse team of Elders, Indigenous patients, healthcare professionals, and healthcare administrators, and Indigenous and non-Indigenous researchers believes that it is vital to do so in a relational way which fosters the inclusion of varying perspectives in the review procedures and interpretation of findings. There is a need to collectively identify and articulate respectful storytelling approaches for patient-orientated research that involves partnerships among Indigenous and non-Indigenous peoples. This collaborative review will inform future directions for decolonizing, patient-oriented research when working with Indigenous communities and their health.

In Canada, the Truth and Reconciliation Commission Calls to Action [8] and the National Inquiry into Missing and Murdered Indigenous Women and Girls Calls for Justice [9] challenge healthcare professionals to include Indigenous knowledge and wellness practices in healthcare. Health and wellness services are most effective when they are designed and delivered in a manner consistent with and grounded in the practices, worldviews, cultures, languages, and values of the distinct Indigenous peoples they serve [9]. However, research has historically been exploitative and conducted with little regard for Indigenous values or priorities, with detrimental consequences for Indigenous peoples’ health [10]. For centuries, Western scientific approaches dismissed Indigenous ways of knowing as incongruent with rigorous research, and colonial perspectives dominated interpretations of Indigenous peoples’ experiences [10–12]. Smith [11] notes that even the word “research” is deeply offensive and traumatic to many Indigenous peoples. When research is conducted from a Western paradigm alone, the findings and resultant policies further entrench colonialism, ignore health and wellness practices of Indigenous peoples, and fail to align with the priorities of Indigenous peoples [12, 13]. Further, non-Indigenous people have committed research atrocities towards Indigenous peoples, such as unethical nutritional experiments and vaccine trials, which has propagated mistrust [12]. Disruption of the dominance of Western ways of knowing within health research requires the advancement of methods that meaningfully involve Indigenous peoples and their worldviews, knowledge, and practices into healthcare [10, 12, 14].

Storytelling is identified as a decolonizing research method which can be grounded in Indigenous epistemologies and foster culturally respectful and responsive research [10]. There is a strong oral history with Indigenous peoples, which involves engaging in relational processes and honoring protocol based on tribal knowledge [12, 15, 16]. Storytelling plays a crucial role within Indigenous communities and can be a powerful form of resistance to colonialism as stories remind people of who they are, where they come from, and what they understand [16–18]. The use of Indigenous languages in storytelling engages people, elicits emotions, results in a greater meaning, and promotes the health and wellness of communities. Storytelling research approaches are widely acknowledged as important methods in Indigenous research and can challenge the dominance of Western worldviews [18]. Stories hold holistic knowledge and situate this knowledge in relationships and place; thus, they provide a culturally nuanced way of knowing and a legitimate form of understanding complex phenomenon related to health [18]. As well, as a form of art-based inquiry, storytelling can open up alternative avenues for expressions of life experiences [19–21]. The arts can engage people physically, emotionally, mentally, socially, and spiritually and provide a vessel for holistic knowledge, which is harmonious with Indigenous values and history. There are numerous examples of storytelling in Indigenous health research which demonstrate its potential for illuminating Indigenous knowledge and practices [5, 15, 22–25]. For example, researchers have used storytelling in a participatory action research project to engage with the community and understand Inuit peoples’ experiences of living with diabetes [22]. Digital storytelling, which involves the creation of a 3–5-min video using multimedia materials (i.e., narrative, photographs, music) to tell a personal story, is increasingly being used in Indigenous health research [26, 27]. Fontaine, Wood, Forbes, and Schultz [5] used digital storytelling to collaborate with First Nations’ women with experiences of a heart condition to understand concepts, language, and experiences about their heart health. Other members of this current research team found a digital storytelling workshop to be instrumental in engaging with First Nations’ women about their experiences with breast cancer [28].

Storytelling methods come with an ethical obligation to ensure that Indigenous peoples and their stories are not exploited [16, 18]. Archibald and colleagues [4] describe the importance of researcher preparation to become story-ready—to learn the nature and protocols for using Indigenous stories respectfully. Stories need to be understood within the context that they are created and with an appreciation of the underlying epistemological assumptions to avoid problematic interpretations and harm infliction. Cunsolo Willox warns that if used without socio-cultural and personal sensitivity, storytelling can “reify, objectify, essentialize, and/or further marginalize individuals and communities” and “allow non-Indigenous researchers to say what they want to say” ([15], p. 129-130). Indigenous health research is often carried out by academics associated with universities, institutions dominated by Western worldviews [18]. Thus, it is critical to understand how the guardianship and interpretation of participants’ stories are occurring within research settings [18].

As the body of research incorporating storytelling as a method in Indigenous health research grows, a critical review aimed to advance our ways of understanding this decolonizing research method is imperative. Our liaison librarian for Indigenous health (JL) searched key databases and found no current or planned review on storytelling as a method in Indigenous health research. This protocol outlines how our team of Indigenous and non-Indigenous researchers, and Indigenous patients, Elders, healthcare professionals, and healthcare administrators, will conduct a critical, participatory scoping review [6, 7] of the use of storytelling in Indigenous health research. Based on our findings, we will then develop a framework for the respectful use of storytelling in health research that involves partnerships of Indigenous and non-Indigenous people. Our work is critical to elevating storytelling within health research and producing evidence located within Indigenous worldviews which can better inform healthcare services and delivery. As well, our project will promote patient-engaged research as our team includes Indigenous patients; together, we will critically reflect upon storytelling methods to facilitate more comprehensive, respectful, and relevant future research projects employing storytelling methods [22].

### Theoretical perspectives guiding our work

The concept of two-eyed seeing will guide our review [29, 30]. A two-eyed seeing perspective proposes that both Indigenous and Western ways of knowing are important for building upon existing knowledge [29, 30]. In this way, knowledge is known to be rooted in a *both/and* perspective rather than an *either/or* perspective, and as such, each worldview is respected as a legitimate source of understanding [29]. As we aim to develop respectful research partnerships between Indigenous and non-Indigenous team members, this approach will enable us to come together as an interdisciplinary research team who will lend distinctive, valuable perspectives to the study. These perspectives will guide the research process, interpretation of findings, and knowledge dissemination.

The Strategy for Patient-Oriented Research Patient Engagement Framework and the Capacity Development Framework will also inform our project [31, 32]. The Patient Engagement Framework prioritizes patients as active partners in research. Key principles for patient engagement are described as inclusiveness; support for patients to ensure full contribution; mutual respect between researchers, clinicians, and patients; and a commitment to “co-build” throughout the research process. This framework has supported the inclusion of Elders, patients, administrators, and healthcare professionals early and throughout the research process and also guides the nature of our partnerships through its patient engagement principles. The goal of capacity development in patient-oriented research is to build training for patient-oriented research, mentoring, and career support into the research project; the Capacity Development Framework serves as a tool to guide this process. Our team will prioritize the ongoing development of a supportive and collaborative research environment for patients, clinicians, trainees, and researchers at multiple career stages.

### Review purpose

Our purpose is to identify and examine how storytelling has been used as a method in Indigenous health research on Turtle Island [33] (i.e., Canada and the USA), Australia, and Aotearoa (New Zealand).

## Methods

The review protocol has been registered within the Open Science Framework database (https://osf.io/rvf7q) and is being reported according to the reporting guidance provided in the Preferred Reporting Items for Systematic Reviews and Meta-Analyses Protocols (PRISMA-P) statement (see checklist in Additional file 1) [34, 35]. The PRISMA Extension for Scoping Reviews (PRISMA-ScR) checklist will structure the reporting of our review [36]. Guided by two-eyed seeing [29, 30], our design will include both Western and Indigenous research approaches. We will conduct a scoping review of research articles that used storytelling as a method in Indigenous health research. A scoping review is a type of knowledge synthesis, emerging from Western ways of knowing, that aims to map “the key concepts underpinning a research area and the main sources and types of evidence available.” [7]^p.21^ Some argue that scoping reviews need to move towards more reflexive and subjective engagement with literature and that they can become critical methodologies if researchers engage in an “unpacking of a problematic that situates the work historically and methodologically.” [10] ^p. 178^ Bassett and McGibbon adapted Arksey and O’Malley’s scoping review method to develop a critical participatory and collaborative scoping review approach, which we will use for our work [6, 7]. Through our Relational Network comprised of Elders, Indigenous patients, healthcare professionals, healthcare administrators, and Indigenous and non-Indigenous researchers, we will map and critique the literature. Our Relational Network will be involved in a collaborative level of engagement throughout all review stages to provide expertise that will shape the scoping review procedures, interpret and critically reflect on this body of work, and guide knowledge translation strategies [6, 10]. Although a scoping review is a Western methodology, we have taken a decolonizing approach to the research and will include ways of knowing that are grounded in Indigenous perspectives such as including traditional practices in our work together, paying careful attention to relational knowing, engaging in collective interpretation and critical reflection with a team of Indigenous and non-Indigenous people, and giving back to the community [10, 18].

Our scoping review will follow six iterative stages: (1) refining the research objectives; (2) identifying relevant studies; (3) study selection; (4) charting the data; (5) collating, summarizing, and reporting the results; and (6) consultation [6]. This fifth stage will also involve co-creating a framework for the respectful use of storytelling in Indigenous health research. Scoping reviews often include a sixth discrete stage of stakeholder consultation, but our participatory approach will be a more intensive, comprehensive collaboration woven throughout all five study stages as academic researchers co-create this work with Elders, and Indigenous patients, healthcare professionals, and healthcare administrators [7].

### Stage 1: Identification of research objectives

Our broad research objectives define our scope of inquiry and will act as a flexible roadmap to guide the review. Our research objectives are to (1) determine the extent, range, and nature of storytelling as a method within Indigenous health research; (2) identify exemplary practices and/or problematic omissions in the respectful use of storytelling in Indigenous health research; (3) develop a framework for the respectful use of storytelling as a method in Indigenous health research to guide future patient-orientated research involving Indigenous and non-Indigenous peoples; and (4) achieve the first three objectives in a participatory and collaborative way through relationship and dialogue with our Relational Network of diverse team members (Elders, Indigenous patients, healthcare professionals, and healthcare administrators, and Indigenous and non-Indigenous researchers) [6]. Indigenous “patients” involved in this review are research team members who have been patients of the healthcare system and also have been previously involved in a storytelling research study. Thus, each “patient” will bring perspectives from both experiences. We will refine our research objectives (1–3) through our Relational Network discussions. Key concepts of our objectives (e.g., storytelling, Indigenous, research) are defined in the next section in which we describe our inclusion and exclusion criteria.

### Stage 2: Identifying relevant studies

Our Relational Network will meet to develop a search strategy regarding inclusion criteria (e.g., storytelling definition), keywords, and literature sources.

#### Search methods

To identify relevant studies, we will conduct a systematic search with keywords, medical subject headings, and/or subject headings combined with Boolean terms and adapted to the syntax in specific databases. Our search strategy and key search terms will be informed by our inclusion criteria, Relational Network discussions, and a preliminary literature review of storytelling and its variants. See Additional file 2 for a search strategy example. Our expert Indigenous health liaison librarian (JL) will systematically search electronic databases and the gray literature for potentially eligible studies [37]. Databases to be searched for academic journal articles will include Scopus, MEDLINE, Embase, EBSCOhost (including CINAHL, AgeLine, Academic Search Complete, Bibliography of Native North Americans, and Canadian Reference Centre), and PsycINFO. Additional searching for core journals in Indigenous health will be done if they are not indexed in MEDLINE, CINAHL, or Embase. As well, we will use snowball sampling and search the reference lists of all included articles and perform a Scopus forward reference search. The gray literature search will include carrying out advanced searches in Google, reviewing the content on relevant websites, and searching other sources recommended by our Relational Network. Unindexed or gray literature will be included if it meets the criteria for inclusion, and the criteria for dates will arise from our iterative review process.

#### Inclusion/exclusion criteria

We will include qualitative primary studies and the qualitative component of mixed-methods primary studies that use storytelling methods during the research process. The studies can include, but will not be limited to, qualitative research designs such as Indigenous methodologies, participatory action research, phenomenology, grounded theory, ethnography, interpretive description, and narrative inquiry. Our inclusion criteria address the key ideas in our review purpose regarding the populations, contexts, and concepts of the included studies. Study participants will be children, adolescents, or adults who are Indigenous peoples, or their healthcare professionals, residing on Turtle Island [33] (i.e., Canada and the USA), Australia, or Aotearoa (New Zealand). We will include these places as they share similar experiences with colonization and notable health disparities among Indigenous peoples [38]. Although there is no widely accepted definition, we define Indigenous populations as “communities that live within, or are attached to, geographically distinct traditional habitats or ancestral territories, and who identify themselves as being part of a distinct cultural group, descended from groups present in the area before modern states were created and current borders defined.” [39] ^(para. 1)^ The healthcare context will encompass a research focus on health phenomena or a study conducted by healthcare professionals. We define the concept of storytelling as participant-driven and created story-centered narratives [15]. Storytelling approaches can include, but are not limited to, sharing circles that invite a story [18], digital storytelling [5], yarning as a conversational process [25], Pūrākau [40], and storytelling in narrative inquiry [23]. A more detailed list of relevant storytelling approaches and terminology will be gathered through our Relational Network discussions and our literature review. Studies with interview-based narratives alone, that in which an interview guide fragments the participants’ story, will be excluded [15, 18]. If storytelling is used solely as a therapeutic or pedagogical intervention, it will also be excluded. We will exclude articles not available by full text or in English, due to the time and cost of acquiring and translating articles.

### Stage 3: Study selection

We will export search results to Covidence [41], a web-based software platform for screening/data extraction, and remove duplicate citations. Two review team members will independently assess the retrieved titles and abstracts against our inclusion criteria and include or exclude them based on this assessment. All potentially relevant articles will be read in full and evaluated for eligibility by two reviewers (see Additional file 3), and reasons for exclusion will be noted. Disagreements at either stage will be resolved through discussion or by consultation with a third reviewer or the research team. We will ensure consistency and rigor by randomly selecting 20 articles which will be independently screened by the two reviewers involved in the screening process; kappa’s co-efficient will be calculated to measure inter-rater agreement and address issues with screening processes.

### Stage 4: Charting (extracting) the data

In-depth Relational Network discussions will be critical for this stage, as what data is collected is influenced by one’s worldview and epistemological assumptions, and subsequently shapes the interpretation of findings. We have collaboratively developed an initial data extraction form (see Additional file 4), which considers data surrounding the characteristics of participants, community members, and researchers; self-location of and relationships between participants, community members, and researchers; setting; study purpose and research question(s); theoretical/epistemological underpinnings; methodology and methods; description of storytelling method; types of stories told; stated benefits and challenges of using storytelling; cultural protocols employed surrounding storytelling; ethical considerations; Indigenous peoples’ role in the interpretation of stories; initiatives to give back to the community; knowledge translation approaches; and Indigenous peoples’ role in creating/checking the representation of shared stories. Once our study is in process, we will review this list and revise it as necessary. We will pilot our data extraction form with five studies and will then review and revise it. One reviewer will extract the data from the included articles and another will check it. Although critically appraising the strength of the studies with a standardized quality appraisal tool is not an expectation in a scoping review, we will employ the Spectrum of Engagement Tool [42] (see Additional file 5) to evaluate the level of engagement with Indigenous peoples in the included research articles.

### Stage 5: Collating, summarizing, and reporting the results

To collate and summarize the data, we will engage in an iterative data analysis process. First, we will describe our included studies (e.g., description of settings and participants). This description will include identifying and cataloging the array of storytelling research methods. Two reviewers will independently conduct a thematic analysis of the extracted data to address research objectives one and two, and through an iterative team data analysis process, we will refine our findings. As part of this process, we will code and note patterns of exemplary practices of respectful approaches to storytelling and also notable omissions. Archibald’s [4] storytelling ethical principles of respect, responsibility, reverence, and reciprocity will inform this analysis. We will also examine the Spectrum of Engagement [42] assessments for all included studies. Finally, to address research objective three, our analysis will involve critically reflecting on (1) how storytelling has been used in Indigenous health research and (2) how it could be used in future studies. We will co-create a framework with strategies to guide the respectful use of storytelling as a method in Indigenous health research involving Indigenous and non-Indigenous peoples.

Our reporting of findings will encompass integrated knowledge translation and end-of-grant dissemination [43]. Congruent with integrated knowledge translation, academic researchers and knowledge users will work collaboratively throughout the research process in our Relational Network (as outlined below) and seek guidance from a stakeholder advisory group comprised of representatives from key community organizations. We will meet once with the advisory group to introduce the work and garner their input into our review, and once near the completion of the review to plan dissemination strategies and future research. This inclusion of knowledge users and stakeholders in the research process will increase the relevance of findings, inform effective dissemination strategies, and move the findings into practice. Our end-of-grant plan will be multi-faceted and effectively communicate results to a range of knowledge users to impact practice and policy. An important focus will be sharing our findings with Indigenous groups with whom we have relational connections. Our plan includes conventional approaches, such as local, national, and international presentations and publishing the review findings and resulting framework in open-access journals, as well as innovative, creative knowledge translation activities. For instance, to share our research findings online and in presentations, we will create a short video to share findings in an engaging, accessible manner with the public, practitioners, policy-makers, and academics [26, 27]. In addition, we will develop a visual infographic which will be used to disseminate our framework for respectful use of storytelling in Indigenous health research in presentations, publications, and within social media (e.g., Twitter).

### Stage 6: Gathering with our relations

Foundational to this work is establishing respectful and authentic relationships within our diverse team so that we can engage and learn from each other. While the sixth stage of a scoping review typically involves consulting stakeholders, our participatory and patient-engaged approach has woven these processes throughout stages 1 through 5. To facilitate meaningful Relational Network engagement and discussions in each stage of the scoping review, we will use numerous strategies. We will engage in an Indigenous ceremony to launch the project and plan to hold four gatherings throughout the project. These days will include a talking circle in the morning with our Elders, patients, healthcare professionals, and healthcare administrators, a lunchtime feast for all team members, and a 2-h meeting with the academic research team in the afternoon. If an Elder, patient, healthcare provider, or administrator wishes to come to the afternoon meeting, they will be welcome to do so. Before the gatherings, we will seek guidance from an Elder to develop the agenda and appropriate protocols.

The morning meetings will be facilitated by an Elder and an academic research team member and be planned so that these discussions guide and inform the scoping review during key stages. An Elder will begin the gatherings with an invocation and welcome. At each meeting, we will hold a talking circle about storytelling (e.g., the meaning of storytelling, team members’ experiences with storytelling, the language used in relation to storytelling, cultural protocols surrounding storytelling) as well as focus on specific components of the scoping review. *The first gathering* will focus on refining the scoping review objectives and developing the search strategy (stages 1 and 2). There will also be an orientation session for patients about their crucial role in this work. *The second gathering* will focus on refining the search strategy and identification of relevant studies and creating the data extraction form (stages 2, 3, and 4). *The third gathering* will focus on reviewing the extracted data to date, refining the data extraction process, and discussing the preliminary review results and interpretation of findings (stages 4 and 5). *The fourth gathering* will focus on discussing the review results and interpretation, imagining how storytelling could be used in future research work based on patient-oriented priorities, co-creating a framework for the respectful use of storytelling in Indigenous health research, and planning the dissemination of our work (stage 5). The afternoon meetings will focus on the scoping review procedures and updates and will provide an opportunity to elicit input from the academic research team members regarding the review work. If a team member is not able to attend in-person, or we are not able to meet in-person due to the COVID-19 pandemic, we will arrange to meet by video or plan individual phone/in-person meetings. In addition, the principal applicant will send regular email updates to the team and meet one-on-one as desired.

### Ethical considerations

We recognize and respect the principles of Indigenous ownership, control, access, and possession of research as crucial to self-determination and doing ethical research [44, 45]. Our team has employed a shared governance model by creating the Relational Network, and these team members have been involved in planning this study and will be involved in all stages of the research described earlier. Our diverse team will guide the dissemination of this work, with a focus on sharing the findings and the framework freely with Indigenous communities and organizations. We will develop accessible knowledge translation strategies such as a short video, a visual infographic, and open-access publications, which can be easily accessed and shared by Indigenous groups and partners. Of note, we will be identifying and analyzing accessible pre-existing research reports and will not be recruiting new study participants, collecting new data, or presenting primary research findings, and thus, a research ethics board approval is not required. We anticipate that the appropriate ethics review boards will have approved all included studies and will document this on the data extraction form.

## Discussion

There has been an exploitative history of research with, on, and for Indigenous peoples, with an accompanying cry for the decolonization of health research [12]. Given the importance of storytelling for Indigenous peoples and the need for respectful research approaches, there is a clear need to elevate storytelling as a research method and to provide guidance on respectful ways to use storytelling when conducting research with teams of Indigenous and non-Indigenous peoples. A research team with a synergistic and broad mix of substantive, methodological, and experiential expertise will conduct this critical participatory scoping review on storytelling as a method in Indigenous health research, which will (1) develop a deeper understanding of the extent, range, and nature of storytelling as an Indigenous health research method; (2) uncover and challenge colonial ideas within this body of health research; and (3) propose guidance for the respectful use of storytelling in research [10]. Any amendments to this protocol made when conducting the review will be noted in the master protocol document and reported and explained in the final review report.

There are several potential operational issues and limitations of this review. As this is a participatory, scoping review which is being launched amid a global pandemic, public health guidelines will need to be taken into consideration and plans to gather may need to be adapted accordingly. To mitigate a negative impact on our review findings, we will consult with our Elders for guidance regarding how to best gather together and engage in Indigenous ceremony if we need to adapt our plans. As well, several review team members have a history of working effectively together to navigate research challenges. Another challenge will be in determining how the term “storytelling” is being used in the retrieved articles, as qualitative researchers frequently use the words “stories,” “story,” or “narrative” to describe a semi-structured interview which does not involve a storytelling method. We will discuss this as a team and determine how to apply our inclusion criteria in these situations. Limitations related to the source of evidence include the possibility of missed studies due to unclear indexing of articles. To decrease the chance of omissions occurring, we have an Indigenous Health Liaison Librarian on our research team who will guide the literature search. We are also excluding articles not published in English, due to feasibility, but may miss some meaningful storytelling studies because of this decision. Lastly, consistent with scoping review procedures, our purpose is not to conduct formal quality appraisals. Thus, our insights regarding the overall quality of the included articles will be limited to descriptions of noted variations. However, we have decided to formally assess one important quality indicator which strongly aligns with our review purpose, that of the level of engagement with Indigenous peoples in the research project, which will be measured with the Spectrum of Engagement Tool [42].

Our scoping review protocol also has significant strengths. Collaborating with Elders, Indigenous patients, administrators, and healthcare professionals will result in a particularly rich and nuanced understanding of this decolonizing research method and will create spaces for diverse voices to inform healthcare research and practices [29, 30]. The non-Indigenous team members will also have rich opportunities to learn about Indigenous ways of knowing and being from Indigenous team members. Partnerships developed amongst patients, health-care professionals, policymakers, and Indigenous and non-Indigenous researchers, along with our review findings, will serve as essential precursors to future research projects employing storytelling as a decolonizing and patient-oriented research method. Consistent with a two-eyed seeing approach [29, 30], our ultimate goal is advancing evidence which meaningfully includes Indigenous perspectives, practices, and priorities into the Canadian healthcare systems to impact health and wellbeing and benefit Indigenous communities. Paying careful attention to how this knowledge is elucidated and where it is grounded is critical to pursuing this worthy goal.

## Supplementary information

**Additional file 1.** PRISMA-P 2015 Checklist.

**Additional file 2.** Search Strategy for MEDLINE.

**Additional file 3.** Verification of Eligibility Form.

**Additional file 4.** Data Extraction Form.

**Additional file 5.** Spectrum of Engagement Tool [42].

## Data Availability

Not applicable.

## Abbreviations

PRISMA-ScR: Preferred Reporting Items for Systematic Reviews and Meta-Analyses extension for Scoping Reviews Checklist
COVID-19: Coronavirus disease of 2019
