# Supplementary material for: Elevating the uses of storytelling approaches within Indigenous health research: a critical and participatory scoping review protocol involving Indigenous people and settlers
Source: Syst Rev. 2020 Nov 4;9:257. doi: 10.1186/s13643-020-01503-6 (PMC7640994; doi:10.1186/s13643-020-01503-6)
Supplement: Supplementary file 2 — Additional file 2. Search Strategy for MEDLINE. [file 13643_2020_1503_MOESM2_ESM.docx]

**Search Strategy for MEDLINE**

| 1 exp indigenous peoples/ or american native continental ancestry group/ or alaska natives/ or indians, north american/ or inuits/ or Oceanic Ancestry Group/  2 (Indigenous or First Nations or First Nation or Inuit* or metis or Aboriginal or Native American* or alaska native* or (Alaska* adj1 Native*) or Ojibw* or Cree or Athapaskan or Athabaskan or Athabascan or Saulteau* or Wakashan or Dene or Inuk or Tlicho or Haida or Ktunaxa or Tsimshian or Gitsxan or Nisga'a or Haisla or Heiltsuk or Oweenkeno or Kwakwaka'wakw or Nuu chah nulth or Tsilhqot'in or Dakelh or Wet'suwet'en or Sekani or Dunne-za or Dene or Tahltan or Kaska or Tagish or Tutchone or Nuxalk or Salish or Stl'atlimc or Nlaka'pamux or Okanagan or Sec wepmc or Tlingit or Anishinaabe or Anishinabe or Blackfoot or Nakoda or Tasttine or TsuuT'ina or Gwich'in or Han or Tagish or Tutchone or Algonquin or Algonkian or Nipissing or Kahnawake or Mohawk* or Cherokee or Potawatomi or Innu or Maliseet or Mi'kmaq or Micmac or Passamaquoddy or Haudenosaunee or Cayuga or Dakota or Lakota or Navajo or Zuni or Hopi or Oneida or Onodaga or Seneca or Tuscarora or Wyandot or Indigeneity or Nunavut* or Iqaluit* or Nunavummiut or Kitikmeot or Kivalliq or qikiqtani or Baffin or Kuujjuaq or Inuvialuit or Nunavik or nunavtsiavut or Inupiat or inupiaq or yupik or Yellowknife or northwest territories or Yukon or Whitehorse or Fairbanks or quajigiartiit or eskimo* or maori* or torres strait island* or koori or goori or murri or nyoongah or Nyoongar or Noongar or Nyunga* or koorie or yolngu or Anangu or palawa or nunga or Ngarrindjeri or murray island or mer island or american Indian* or aborigine* or indigen* or Hawaii* or ha waii* or Menominee or Ahousat or Apache tribe or Arapahoe or Bella Coola or Paiute or Shoshone or Blackfeet or Cherokee or Cheyenne or Sioux or Siouan or Choctaw Indian* or Choctaw Nation* or Comanche nation or Kootenai Tribes or Dogrib or Flathead Nation or Flathead Reservation or Havasupai or Pima or Tohono O'odham).mp.  3 Health Services, Indigenous/  4 1 or 2 or 3  5 (story* or stories or oral tradition* or oral histor* or biograph* or autobiograph* or talking circle* or sharing circle* or talking stick or yarn* or talkstory or talk story or re storying or re membering or narrative inquiry or narrative enquiry or (digital adj3 narrative) or narrative data or narrative therapy or narrative medicine or conversational method* or talking cure* or blood memory or ways of knowing or ways of seeing or ways of doing or ways of feeling or whakatauki or whakarongo or purakau or arts based).mp.  6 4 and 5  7 limit 6 to english language |
| --- |
