# Supplementary material for: Elevating the uses of storytelling approaches within Indigenous health research: a critical and participatory scoping review protocol involving Indigenous people and settlers
Source: Syst Rev. 2020 Nov 4;9:257. doi: 10.1186/s13643-020-01503-6 (PMC7640994; doi:10.1186/s13643-020-01503-6)
Supplement: Supplementary file 3 — Additional file 3. Verification of Eligibility Form. [file 13643_2020_1503_MOESM3_ESM.docx]

**Verification of Eligibility Form**

| Author and year: |
| --- |
| Title: |
| Reviewer: |
| Population: Indigenous peoples, or their healthcare professionals, residing on Turtle Island (North America), Australia, or Aotearoa (New Zealand). We define Indigenous populations as “communities that live within, or are attached to, geographically distinct traditional habitats or ancestral territories, and who identify themselves as being part of a distinct cultural group, descended from groups present in the area before modern states were created and current borders defined”.  Yes/No  Comments: |
| Phenomenon/intervention of interest: Use of storytelling (participant-created story-centered narratives) at any point in the Indigenous health research process  Yes/No  Comments: |
| Context: The research was conducted in healthcare context, which can encompass focusing on health-related phenomenon or research conducted by healthcare professionals.  Yes/No  Comments: |
| Study design: All qualitative and mixed-methods study designs will be included.  This study is original qualitative or mixed-methods research: Yes/No  The study is original qualitative research:  Qualitative design: _______________________  The study is original mixed-methods research:  Qualitative design: ______________________  Comments: |
| If you answer yes to all of the above questions, continue to data extraction. If not, then exclude the paper.  Yes/No  Comments: |
