# Supplementary material for: Elevating the uses of storytelling approaches within Indigenous health research: a critical and participatory scoping review protocol involving Indigenous people and settlers
Source: Syst Rev. 2020 Nov 4;9:257. doi: 10.1186/s13643-020-01503-6 (PMC7640994; doi:10.1186/s13643-020-01503-6)
Supplement: Supplementary file 4 — Additional file 4. Data Extraction Form. [file 13643_2020_1503_MOESM4_ESM.docx]

**Data Extraction Form**

| Reviewer | | | Date | |
| --- | --- | --- | --- | --- |
| **Storytelling in Indigenous Health Research: Extraction Tool For Included Studies** | | | | |
| **Publication Information** | | | | |
| Title | | Author(s) | | |
| Date | Journal/Publication Status | | | |
| Country | | | | |
| **Data Extraction Component** | | | | **Location in Text** |
| **Setting** (i.e. clinical or community setting, geographical location) | | | |  |
|  | | | |  |
| **Participants’ Characteristics and Self-location** | | | |  |
|  | | | |  |
| **Community Members’ (i.e. stakeholders) Characteristics and Self-location** | | | |  |
|  | | | |  |
| **Researchers’ Characteristics and Self-location** | | | |  |
|  | | | |  |
| **Relationship between Participants, Community Members, and Researchers** | | | |  |
|  | | | |  |
| **Theoretical/Epistemological Underpinnings** | | | |  |
|  | | | |  |
| **Study Purpose and Research Questions** | | | |  |
|  | | | |  |
| **Methodology** | | | |  |
|  | | | |  |
| **Methods** | | | |  |
|  | | | |  |
| **Description of Storytelling Method and Types of Stories Told** | | | |  |
|  | | | |  |
| **Stated Benefits of Using Storytelling** | | | |  |
|  | | | |  |
| **Stated Challenges of Using Storytelling** | | | |  |
|  | | | |  |
| **Cultural Protocols Employed Surrounding Storytelling** | | | |  |
|  | | | |  |
| **Ethical Considerations** | | | |  |
|  | | | |  |
| **Indigenous Peoples’ Role in Interpretation of Stories** | | | |  |
|  | | | |  |
| **Initiatives to Give Back to the Community** | | | |  |
|  | | | |  |
| **Knowledge Translation Approaches** | | | |  |
|  | | | |  |
| **Indigenous Peoples’ Role in Creating/Checking the Representation of Shared Stories** | | | |  |
|  | | | |  |
