# Supplementary material for: Elevating the uses of storytelling approaches within Indigenous health research: a critical and participatory scoping review protocol involving Indigenous people and settlers
Source: Syst Rev. 2020 Nov 4;9:257. doi: 10.1186/s13643-020-01503-6 (PMC7640994; doi:10.1186/s13643-020-01503-6)
Supplement: Supplementary file 5 — Additional file 5. Spectrum of Engagement Tool [42]. [file 13643_2020_1503_MOESM5_ESM.docx]

**Spectrum of Engagement Tool^39^**

|  | **NONE** | **INFORM** | **CONSULT** | **INVOLVE** | **COLLABORATE** | **LEAD** |
| --- | --- | --- | --- | --- | --- | --- |
| Goal of Indigenous involvement in research | No evidence of Indigenous involvement. | To provide Indigenous peoples with information about the research project, and assist them in understanding the research problem and/or findings. Examples: meetings with Indigenous community members or Elders to inform about the research project. No control in the research process. | To obtain Indigenous feedback on the research process, data analysis, research findings, and/or decisions to implement research findings. Examples: meetings with Indigenous community members or Elders to get feedback on the research project. No control in the research process. | To work directly with Indigenous peoples throughout the research process to ensure that Indigenous concerns and aspirations are understood and considered.  Examples: Indigenous advisory group; community members as data collectors. No control in the research process. | To ***partner with*** Indigenous peoples in each aspect of the research process, including the research design, execution, data analysis, interpretation and dissemination of findings. Examples: part of the research team; role in shaping the research study. Some control in the research process. | To have Indigenous peoples ***lead*** each aspect of the research process, including the research design, execution, data analysis, interpretation and dissemination of findings. Examples: leading methodological decisions, first authorship. Full control in the research process. |
